# Dismantling Barriers to Hepatitis B and Delta Screening, Prevention, and Linkage to Care among the PWUD Community in Philadelphia

**DOI:** 10.3390/v16040628

**Published:** 2024-04-18

**Authors:** Beatrice Zovich, Catherine Freeland, Holly Moore, Kara Sapp, Anousha Qureshi, Rachel Holbert, Jason Zambrano, Daljinder Bhangoo, Chari Cohen, Richard W. Hass, Amy Jessop

**Affiliations:** 1 Hepatitis B Foundation, Doylestown, PA 18902, USA; 2 HepTREC at Prevention Point Philadelphia, Philadelphia, PA 19134, USA; 3 Jefferson College of Population Health, Thomas Jefferson University, Philadelphia, PA 19107, USA

**Keywords:** hepatitis B, hepatitis delta, people who use drugs, people who inject drugs, harm reduction, population health, viral hepatitis, liver cancer

## Abstract

The prevalence of hepatitis B and delta viruses (HBV/HDV) among people who use drugs (PWUD) remains largely unknown. In the context of one Philadelphia-based harm reduction organization (HRO), this study aimed to assess HBV/HDV prevalence and facilitate linkage to care. Participants completed a demographic HBV/HDV risk factor survey and were screened for HBV and reflexively for HDV if positive for HBV surface antigen or isolated core antibody. Fisher’s exact tests and regression were used to understand relationships between risks and HBV blood markers. Of the 498 participants, 126 (25.3%) did not have hepatitis B immunity, 52.6% had been vaccinated against HBV, and 17.9% had recovered from a past infection. Eleven (2.2%) participants tested positive for isolated HBV core antibody, 10 (2.0%) for HBV surface antigen, and one (0.2%) for HDV antibody. History of incarceration was associated with current HBV infection, while transactional sex and experience of homelessness were predictive of previous exposure. This study found high rates of current and past HBV infection, and a 10% HBV/HDV co-infection rate. Despite availability of vaccine, one quarter of participants remained vulnerable to infection. Findings demonstrate the need to improve low-threshold HBV/HDV screening, vaccination, and linkage to care among PWUD. The study also identified gaps in the HBV/HDV care cascade, including lack of point-of-care diagnostics and lack of support for HROs to provide HBV services.

## 1. Introduction

More than 15.6 million people around the world inject drugs, and many are living with serious comorbidities [1]. In the United States, over 6.5 million people have injected drugs in their lifetimes [2]. People who use drugs (PWUD), and particularly people who inject drugs (PWID), are susceptible to blood-borne viruses, as unsafe injection practices, such as using unsterile needles and re-using syringes, increase one’s risk of exposure to viral infections. Other factors, such as unsafe sexual practices, including transactional sex (typically necessitated by social and financial circumstances), as well as homelessness and a history of incarceration, can also heighten the risk of exposure to viral infections in this population [3,4,5,6,7,8]. The most common of these exposures are to human immunodeficiency virus (HIV) and hepatitis B, C, and delta [9].

Hepatitis B is one of the most prevalent serious liver infections in the world. It is caused by the hepatitis B virus, which attacks and injures the liver. A hepatitis B test typically involves a triple-panel test, consisting of hepatitis B surface antigen (HbsAg), which indicates an active hepatitis B infection; hepatitis B surface antibody (HBsAb), which indicates immunity to the virus; and hepatitis B core antibody total (HBcAb), which indicates past exposure to the virus. Hepatitis delta (also known as hepatitis D) is the most severe form of viral hepatitis and can cause rapid progression to advanced liver disease [10,11]. Only those living with or at high risk for hepatitis B can contract hepatitis delta, and hepatitis delta is estimated to impact approximately 5–10% of individuals living with hepatitis B [12]. Superinfection (infection with hepatitis D after an individual has already acquired hepatitis B) is the most common occurrence of hepatitis delta coinfection and leads to more severe liver disease than a chronic hepatitis B infection alone. Up to 90% of superinfected individuals will develop chronic infections of both hepatitis B and delta, of which approximately 70% will progress to cirrhosis, compared to 15–30% of those infected only with the hepatitis B virus [12]. Up to 30% of people living with chronic hepatitis B infection, and as many as 70% of people living with both viruses, will die prematurely from cirrhosis or liver cancer [10,11].

Testing levels for hepatitis B (HBV) and hepatitis delta (HDV), especially among PWUD, are low, and the data are not robust, but the estimated prevalence of HBV among PWUD ranges between 4% and 12% in the United States [1,13]. PWUD are considered to be a group at high risk for hepatitis B and hepatitis delta virus infections and are recommended by the American Association for the Study of Liver Disease (AASLD) to be prioritized for screening [13,14]. Moreover, as of March 2023, all adults in the United States aged 18 years and older are recommended to be screened for hepatitis B at least once in their lifetime [15]. However, systematic screening for HBV and HDV remains limited and is not routinely conducted, leaving both infections frequently underdiagnosed. Screening rates for hepatitis delta specifically are especially low, and the true epidemiological burden is underestimated [11,16]. Screening is often not performed due to low awareness of HDV among healthcare providers and at-risk communities, limited test availability in many commercial laboratories, complicated screening guidelines, and a lack of resources overall [12]. 

Injection drug use is also an indication for vaccination against HBV infection (from which HDV immunity can also be gained, given the dependent nature of HDV upon HBV for survival and replication) [3]. Additionally, as of 2021, the CDC’s Advisory Council on Immunization Practices recommends hepatitis B vaccination for all adults in the United States between the ages of 19 and 59 and for high-risk adults ages 60 and over [17]. Even though PWUD are at high risk of severe illness and poor health outcomes, this population is less likely to seek vaccination against HBV [7,18,19]. 

In order to better understand how best to advance viral hepatitis elimination goals at all levels, from local to global, there is an urgent need to improve HBV and HDV awareness, accurately assess prevalence, and increase prevention and linkage to care in populations that are most at-risk, including PWUD and PWID. It is essential to engage these communities and ensure an uninterrupted cascade of care in the community’s own spaces and terms. The aims of this study were to determine the prevalence of HBV and HDV among members of the PWUD community in Philadelphia and to facilitate connection to HBV vaccination or to HBV or HDV management and care as appropriate. This project was designed to serve as a pilot program for hepatitis B and delta outreach, education, and screening within a harm reduction setting in the United States and to provide a model for the integration of HBV and HDV screening in this population. 

## 2. Materials and Methods

Between August and September 2023, participants seeking services at a harm reduction organization (HRO) in Philadelphia, PA, USA, were invited to participate in a HBV and HDV screening program. Participants were required to be at least 18 years of age, receiving services of any kind at the HRO, able to provide consent for services in English or Spanish, and agreeable to being listed in the electronic medical record system of the harm reduction site at which the screenings were performed. Individuals presenting with visual impairment or who are illiterate were still able to participate via the provision of verbal consent. 

Potential study participants were informed of the study by research staff who explained the project’s purpose and processes and assessed participants for eligibility. Trained staff conducted in-person consent with all prospective participants, fully describing the potential benefits and risks of participation. After signing a consent form, participants completed a routine demographic survey, which included questions about preferred language, services received at the harm reduction site, any previous testing or vaccination received for any type of viral hepatitis, and risk factors for viral hepatitis (Supplementary File S1). The suite of data collection tools known as the Open Data Kit (ODK) was used to capture data from the demographic questionnaire.

One 3 mL sample of blood was drawn on-site from each participant by a licensed phlebotomist. Quest Diagnostics conducted a blood sample screening. Blood samples were assessed using the hepatitis B triple-panel test (hepatitis B surface antigen (HBsAg), hepatitis B surface antibody (HBsAb), and hepatitis B core antibody (HBcAb total)). Reflex testing for hepatitis delta total antibody (anti-HDV) was performed on any individuals testing positive for either HBsAg or isolated HBcAb. All participants were compensated 20 dollars for completing the demographic survey and screening. 

Following screening, participants were encouraged to return to the harm reduction site to receive their results in person from study staff and were given reminder cards with dates, times, locations and staff contact information for collecting results. Participants were incentivized with an additional 15 dollars for returning to review their test results in person. A simplified summary of test results was printed for each participant, along with a letter explaining the test results. Anyone testing positive for HBsAg, isolated HBcAb, and anti-HDV was provided with detailed information, and efforts were made to connect these individuals to appropriate medical care, as well as health insurance and healthcare navigation, wherever possible. The medical care included either hepatology or infectious disease specialty care, depending on which would be of greatest convenience for the specific individual. Those found to have isolated HBcAb were recommended for repeat surface antigen and HBV DNA testing. Those found to be positive for HBsAg and anti-HDV were recommended for HDV RNA testing and evaluation for treatment. Any participants who were found to not have immunity to hepatitis B (and delta), determined by the absence of HBsAg and HBcAb, were encouraged to return to the site on specific dates on which the city health department would be providing hepatitis B vaccines free of charge. Every attempt was made to contact participants who did not receive their results in person, including via mail, telephone, and/or the assigned on-site case manager. Participant test results were also provided to staff at the harm reduction site for inclusion in participants’ electronic medical records. 

A sample size calculation was performed prior to the study, determined by the estimated prevalence of HBV and HDV in Philadelphia within this population, using previous studies conducted locally by the health department and the volume of clients served within this harm reduction organization. The target sample size for screening hepatitis B and delta using a 95% confidence interval, with an estimated population of 30,000 served annually by the harm reduction organization and a 5% margin of error, was approximately 380 people. To account for the expected prevalence of hepatitis delta within this population, the desired sample size was increased to 480 for this study. 

### Data Analysis

Data were extracted from ODK and analyzed using R software (RStudio version 2023.03.0+386) to examine the association between HBV infection status and risk factors for HBV. A statistical model was developed to predict a hepatitis B infection (using HBsAg-positive and HBcAb-positive). Fisher’s exact tests were performed to calculate odds ratios and the significance of variables for HBV blood markers and behavioral risks. Backward stepwise logistic regression was then conducted to identify possible predictors of the outcomes for infection (HBsAg and HBcAb) using R (with library MASS). At each step, variables were added based on *p*-values, and Akaike information criteria (AIC) were used to limit the total number of variables included in the final model. To improve model convergence, variables with greater than 20% missingness were removed. These variables included self-reported history of positive test results and vaccination for hepatitis A and B and a self-reported history of positive test results for hepatitis D. Variables with complete separation were also excluded from consideration. An alpha of 0.05 was utilized for analysis. 

## 3. Results

The study population consisted of 513 individuals. Fifteen individuals were excluded from the analysis due to missing data points, leaving a total sample of 498. Among the study population, 38.4% identified as female (*n* = 191), 61.4% as male (*n* = 306), and 0.2% as nonbinary (*n* = 1). The median age of the study population was 40, with ages ranging from 19 to 76. These and other demographics, including race, are captured in Table 1. Other statistics detailing participants’ engagement in harm reduction services are outlined in Table 2. One hundred twenty-six participants (25.3%) did not have hepatitis B immunity, meaning that they tested negative for hepatitis B surface antigen (HbsAg), hepatitis B surface antibody (HBsAb), and hepatitis B core antibody (HBcAb). Two hundred sixty-two (52.6%) had been previously vaccinated, meaning that they tested positive for HBsAb and negative for HbsAg and HBcAb. Of these, 169 (64.5%) were in the 34–48 year age range, 68 (26%) were 18–33 years of age, 22 (8.4%) were in the 49–63 year age range, and three (1.15%) were 64–78 years of age. Eighty-nine participants (17.9%) had developed immunity through past infection, meaning that they tested positive for HBcAb and HBsAb and negative for HBsAg. Of these, 44 (49.4%) were in the 34–48 year age range, 29 (32.6%) were 49–63 years of age, 15 (16.9%) were 18–33 years of age, and one (1.12%) was in the 64–78 year age range. Eleven individuals had isolated HBcAb (2.21%). Four of these were in the 34–48 year age range, four were in the 49–63 range, two were in the 64–78 year age range, and one was in the 18–33 range. From the study sample, 2% tested positive for HBsAg and HBcAb, indicating a current infection (*n* = 10). Six of these were in the 34–48-year age range, three were 18–33, and one was in the 49–63-year range. One individual was identified as living with hepatitis delta antibody (0.20% of the total sample and 10% of those testing positive for HBsAg). For model development, after removing missing variables and null odds ratios, there were 11 variables considered potential predictors of HBsAg status or history of a previous infection (HBcAb-positive). From the model of all the predictive risk variables, the only one found to be significant was whether an individual had been previously incarcerated (*p* = 0.047, OR = 0.24). Backward stepwise logistic regression was able to confirm only previous incarceration as predictive of HBsAg status (Table 3 and Table 4). Engagement in transactional sex and experiences of homelessness were identified as predictive of HBcAb status. 

In general, HBV vaccine-derived immunity was found to be more prevalent among younger participants, who were born closer to the introduction of HBV childhood vaccine recommendations in the United States. Current drug use was the risk factor most correlated with vaccine-derived immunity (Table 5). More details on the analysis can be found in Supplementary Tables S1–S3. 

A total of 439 (88.2%) returned to receive their test results in person from study staff, and, by the conclusion of the study, 10 participants had received the first dose of the HBV vaccine from health department staff, representing 7.9% of those susceptible and 2% of the total study population.

## 4. Discussion

The rates of current HBV infection in this study were nearly three times greater than those in the general US population. A recent meta-analysis by Wong et al. estimating the prevalence of hepatitis delta infection among adults in the United States in 2022 found an approximate HDV prevalence rate of 3.8% among those living with chronic hepatitis B, a group mainly composed of immigrants [20]. Thus, the observed HDV prevalence rate of 10% found in this study is substantially higher than in the general United States population. 

The risk factors found to be associated with positive HBV status in this study included a history of incarceration, which was associated with the presence of HBsAg, and experiences with homelessness and transactional sexual encounters, which were associated with the presence of HBcAb. Previous literature examining risk factors associated with HBV and HDV status has demonstrated HDV antibody prevalence among PWUD to be positively associated with a longer duration of drug use and resolved hepatitis C (HCV) infection. In one study, PWUD participants who were living with HBcAb were approximately eight times more likely to have an HDV infection if they had a resolved HCV infection than if they were living with chronic HCV. For those testing positive for HBsAg, this difference was twofold [9]. Given the relationship found between positive HBV status and a history of incarceration, findings from this study underscore the importance of viral hepatitis care and prevention within the penal system. The importance of providing vaccines in correctional settings is also evident, given the high proportion of study participants who had previously been incarcerated, and it is critical to incorporate robust vaccination efforts into overall hepatitis B elimination planning.

Because hepatitis B and delta testing require a venous blood draw, the need for a skilled phlebotomist to perform these was essential to the success of this study. Other research has also found the need for venipuncture to be a barrier to performing hepatitis B screening among PWUD, as many members of this community often avoid blood tests due to pain and difficulty [21,22,23]. This demonstrates the value of a hepatitis B and delta point-of-care test, particularly in high-risk settings. Such a test has not yet been approved for use in the United States. Use of a point-of-care test would not only eliminate the discomfort and hardship associated with obtaining blood samples via venous draw but would also allow for much more rapid delivery of results and immediate connection to vaccination or appropriate care, thus significantly reducing the risk of loss to follow-up. Additionally, point-of-care testing would help to mitigate other challenges that presently impede screening in this population, including the need for a physician order and an established relationship with a commercial laboratory. The lack of a point-of-care test only exacerbates the serious problem of missed opportunities to identify individuals living with and susceptible to hepatitis B and delta, thus making effective management of these viruses at the individual and population levels that much more challenging. 

The timely, in-person delivery of test results to participants in this study proved to be highly effective in increasing levels of follow-up and linkage to vaccination. Similar initiatives that included single-visit test-and-treat models of care delivery for hepatitis C in other settings have also found success [24]. Although a return visit was needed for the delivery of results following blood sample collection, this study demonstrated the effectiveness of offering incentives to encourage participants to come back for results and counseling. Directing individuals who were susceptible to HBV to receive first-dose vaccines on-site in parallel with the reception of results was also an effective practice. For the approximately 11% of participants who did not return for test results, effectively communicating results proved to be challenging as many participants did not have updated contact information. Linkage to appropriate care also proved to be difficult in this context. Although every effort was made by study staff to connect participants to needed care, a number of obstacles impeded the achievement of this goal, including the need for participants to meet certain criteria to be eligible to see the infectious disease specialist on-site at the HRO and the challenging logistics involved with making and keeping specialist appointments, which were often located in geographically distant parts of the city. Maintaining contact with this very transient community is also an ongoing challenge, as mentioned above. Additionally, physical health was of low importance for most participants and was superseded by other needs perceived to be more pressing, including housing, food, and avoidance of withdrawal, a finding also demonstrated in previous literature [25]. 

Existing research has found that proactively offering screening, vaccination, and treatment referrals at a centralized site, such as a syringe services program (SSP), rather than referring participants to external settings, increases vaccine uptake and healthcare follow-up within the PWUD population, as this frequently eliminates both financial and transportation barriers [19,26]. A 2019 study by Tressler et al. found that interventions designed to administer accelerated schedule (two-dose) hepatitis B vaccines, alongside services like case management, peer coaching, and hepatitis care coordination, as well as financial incentives, were moderately effective at increasing HBV vaccination completion among PWUD [27]. 

In a 2018 study conducted by the Philadelphia Department of Public Health to better understand susceptibility to hepatitis A and B viruses among PWUD in Philadelphia, 32.6% of study participants were found to be susceptible to hepatitis B. This study also revealed that 43% of individuals tested had evidence of HBV vaccine-derived immunity, and 24.3% had recovered from past HBV infection [5]. These results align closely with the results of the present study but do demonstrate a notable decline in susceptibility and an increase in vaccine-derived immunity and underscore the benefits of coordination and collaboration with public health departments, especially for the provision and administration of vaccines, which was found in the present study to be vital to the successful connection of individuals susceptible to hepatitis B with vaccines. 

Another barrier currently associated with HBV vaccine administration is the need for insurance coverage and copay support. Currently, the CDC only subsidizes adult vaccines for individuals who are uninsured, leaving many who are under-insured to pay out of pocket for expensive costs related to vaccination that their insurance may not cover. Provision of affordable adult vaccines to a much broader expanse of the population is necessary to ensure more widespread coverage and to contribute in a meaningful way toward hepatitis B elimination in the United States. 

The use of reflex testing for hepatitis delta, as was used in this study, has been shown to increase timely detection and linkage to care for people living with the virus, thus reducing liver disease-related mortality [11,28]. The lack of automatic reflex testing on HBsAg-positive blood samples has contributed to hampering overall screening for HDV infection globally. In one 2019 study, only 42% of people with chronic HBV were tested for HDV, and 8% of those people had a positive HDV test result [16]. Other research has found that the implementation of anti-HDV testing in all HBsAg-positive patients increased the number of HDV diagnoses by five times. As HDV is currently underreported, systematic anti-HDV reflex testing can be a method to improve diagnostic rates and linkage to care for hepatitis delta [11]. The expansion of automatic HDV reflex testing on positive HBsAg samples should be explored as an essential tool to better understand HDV population prevalence and to address this dangerous public health threat in keeping with equitable public health principles and practice. To ensure this happens, it is necessary for current HDV screening guidelines from professional societies, such as AASLD, to be updated to encompass this important need. 

Despite their efficacy in serving the PWUD community, HROs and SSPs can face several barriers to patient health education and disease screening, including limited staff availability and the challenge of working with communities that generally lack access to primary care [29]. These study findings exemplify the need for policies and macro-level viral hepatitis elimination programming tailored to PWUD to be comprehensive and address hepatitis in a way that is thoughtful and intersectional with other important health and social justice issues [30]. On a broad systemic level, inappropriate service delivery models and political and financial constraints that impede public health interventions for PWUD, such as harm reduction services in general and testing for chronic diseases, pose a significant barrier to the timely diagnosis of health conditions and linkage to suitable care for this population [4]. Additionally, much of the focus on disease mitigation in this population (and subsequently much of the funding) has been centered around HIV and hepatitis C, evidence for which can be found in a 2023 harm reduction framework published by the Substance Abuse and Mental Health Services Administration, from which mention of hepatitis B is entirely absent (SAMHSA) [31]. It is of particular interest to note that, based on the findings of this study, the HBV prevalence rate at the harm reduction organization at which this study was conducted is comparable to the current prevalence rate of HIV among the organization’s clientele who requested testing, which is also 2%. This rate is in turn higher than the HIV prevalence rate in the general population of the City of Philadelphia in 2021 (the most recent year for which data are available), which was 1.16% [32]. Therefore, a reallocation of funding and resources to integrate hepatitis B and delta in these spaces would be a worthwhile consideration. 

### Limitations 

This study had several limitations. The phrasing of the demographic questions may have contributed to a failure to fully capture some of the behavioral risk factors in which participants engaged. For example, participants were asked if they engaged in unprotected sexual activity in the past six months, but many individuals were in monogamous relationships, and this was unaccounted for in the data collection, thus perhaps assigning inaccurate levels of risk to some participants. Additionally, in this population, participants presented with various levels of mental alertness and, although coherent and able to provide informed consent, were frequently unable to recount information about health and past experiences accurately. Finally, given the fact that vaccines were offered during the study as well to minimize loss to follow-up, the possibility exists that some participants may have falsely tested positive for HBsAg due to the narrow timeframe between receipt of a vaccine dose and testing. Vaccines were only provided on two days during the two-month study period, and although study staff made every effort to direct participants to testing first, the busy and somewhat chaotic nature of the space meant that at least one participant received the vaccine before the test. (This participant’s results were excluded from the final prevalence rate.) This study only captured data on current infections at the time of screening and did not assess differences between acute and chronic infections. Despite this, this study does demonstrate that hepatitis B and delta are being transmitted and prevalent in this community.

The enticement of the financial incentive led to several participants attempting to complete the screening multiple times, which in turn led to difficulties with accurate reporting. The threshold for inclusion in this study was kept purposefully low, and the presentation of identification documentation was not required, as this would have been overly burdensome and precluded the participation of a large proportion of community members. All efforts were made to ensure participation was unique per individual; however, it is possible that some duplication existed towards the end of the recruitment period.

## 5. Conclusions

Hepatitis B and delta viruses remain important and under-prioritized public health concerns among people who use drugs. Despite the long-standing status of PWUD as a group at high risk for hepatitis B and delta, the diagnosis and prevention of both viruses remain low in these communities. This study’s findings indicate that hepatitis B and delta remain prevalent in this population. As this study demonstrates, consistent and robust screening, vaccination, and linkage to care efforts can positively impact health outcomes for this frequently marginalized community. While executing such an initiative can present a unique and complex set of challenges, implementation of certain recommendations, including approval of a hepatitis B point-of-care test, funding and political support for provision of comprehensive healthcare services at HROs, accessible and low-threshold collaboration opportunities with public health departments, adequate insurance coverage for vaccines, and recommendation and uptake of universal HDV reflex testing, can contribute greatly to moving the needle on hepatitis B and delta diagnosis, prevention, and treatment, thus advancing elimination efforts, lowering mortality, increasing quality of life, and centering the health and needs of people who use drugs. 

## Figures and Tables

**Table 1:** Demographic characteristics of study participants in relation to active HBV infection.

Category	HBsAg+		HBsAg−		Total	
	*n*	%	*n*	%	*n*	%
Sex						
Female	4	40%	187	38.3%	191	38.4%
Male	6	60%	300	61.5%	306	61.4%
Non-Binary	0	-	1	0.2%	1	0.2%
Race						
White	6	60%	268	54.9%	274	55.0%
Black	2	20%	117	24.0%	119	23.9%
Hispanic	0	-	55	11.3%	55	11.0%
Asian	0	-	3	0.6%	3	0.6%
Native American	1	10%	3	0.6%	4	0.8%
Multiracial *	1	10%	29	5.9%	30	6.0%
Other	0	-	11	2.3%	11	2.2%
Unreported	0	-	2	0.4%	2	0.4%
Ethnicity						
Hispanic	2	20%	94	19.3%	96	19.3%
Non-Hispanic	8	80%	381	78.1%	389	78.1%
Unknown	0	-	6	1.2%	6	1.2%
Unreported	0	-	7	1.4%	7	1.4%
Age						
18–33	3	30%	108	22.1%	111	22.3%
34–48	6	60%	285	58.4%	291	58.4%
49–63	1	10%	87	17.8%	88	17.7%
64–78	0	-	8	1.6%	8	1.6%
Birthplace						
USA	10	100%	472	96.7%	482	96.8%
Other	0	-	16	3.3%	16	3.2%

* Multiracial indicates self-reporting more than one of the above races listed.

**Table 2:** Study participant self-reported engagement in harm reduction services.

Service	Frequency	% of Total (*n* = 498)
Services at HRO		
Syringe Service Program	340	68.3%
Drug Treatment	73	14.7%
HIV/HCV Testing	265	53.2%
Medical Care	159	31.9%
Other	189	38.0%
None	43	8.6%
Unreported	11	2.2%
Case Manager		
Yes	151	30.3%
No	343	68.9%
Unreported	4	0.8%

**Table 3:** Self-reported risk factor variables and odds ratios for the study sample, and association with active hepatitis B infection.

Category	HBsAg+		HBsAg−		Total		*p*-Value	OR (95% CI)
	*n*	%	*n*	%	*n*	%		
Tattoo							1	0.9 (0.17–8.8)
Yes	8	80%	397	81.4%	405	81.3%		
No	2	20%	90	18.4%	92	18.5%		
Unreported	0	-	1	0.2%	1	0.2%		
Incarcerated *							0.047	0.24 (0.05–1.3)
Yes	5	50%	408	83.6%	413	82.9%		
No	4	40%	80	16.4%	84	16.9%		
Unreported	1	10%	0	-	1	0.2%		
Unprotected Sex							0.46	0.65 (0.15–4.0)
Yes	7	70%	381	78.1%	388	77.9%		
No	3	30%	106	21.7%	109	21.9%		
Unreported	0	-	1	0.2%	1	0.2%		
Transactional Sex							1	1.00 (0.17–4.5)
Yes	3	30%	145	29.7%	148	29.7%		
No	7	70%	336	68.9%	343	68.9%		
Unreported	0	-	7	1.4%	7	1.4%		
Unhoused							0.48	0.60 (0.14–2.96)
Yes	6	60%	346	70.9%	352	70.7%		
No	4	40%	139	28.5%	143	28.7%		
Unreported	0	-	3	0.6%	3	0.6%		
Use Drugs							0.44	0.53 (0.07–24)
Yes	9	90%	459	94.1%	468	94%		
No	1	10%	27	5.5%	28	5.6%		
Unreported	0	-	2	0.4%	2	0.4%		
Receive HRO Services							1	-
Yes	10	100%	433	88.7%	443	89%		
No	0	-	37	7.6%	37	7.4%		
Unreported	0	-	18	3.7%	18	3.6%		
Case Manager							0.50	1.53 (0.31–6.6)
Yes	4	40%	147	30.1%	151	30.3%		
No	6	60%	337	69.1%	343	68.9%		
Unreported	0	-	4	0.8%	4	0.8%		

* Indicates significance.

**Table 4:** Methods of self-reported preferred drug use and association with active hepatitis B infection.

Category	HBsAg+		HBsAg−		Total		*p*-Value	OR (95% CI)
	*n*	%	*n*	%	*n*	%		
Injection							0.16	5 (0.66–223.8)
Yes	8	88.9%	283	61.5%	291	62%		
No	1	11.1%	177	38.5%	178	38%		
Share Needles							0.72	1.2 (0.19–6.5)
Yes	3	37.5%	92	32.5%	95	32.6%		
No	5	62.5%	186	65.7%	191	65.6%		
Unreported	0	-	5	1.8%	5	1.7%		
Inhalation (Oral)							0.17	0.28 (0.03–1.51)
Yes	2	22.2%	231	50.2%	233	49.7%		
No	7	77.8%	229	49.8%	236	50.3%		
Ingestion							0.35	2.6 (0.06–21.0)
Yes	1	11.1%	21	4.6%	22	4.7%		
No	8	88.9%	439	95.4%	447	95.3%		
Inhalation (Nasal)							0.35	2.6 (0.05–21.0)
Yes	1	11.1%	21	4.6%	22	4.7%		
No	8	88.9%	439	95.4%	447	95.3%		

Note. OR and *p*-values reported based on those that use drugs (*n* = 469). Sharing needles OR and *p*-values reported based on those that inject (*n* = 291).

**Table 5:** Self-reported risk factor variables and odds ratios for the study sample, and association with hepatitis B serologic immunity.

Category	Vaccinated		Not Vaccinated		Total		*p*-Value	OR (95% CI)
	*n*	%	*n*	%	*n*	%		
Tattoo								
Yes	213	81.3%	192	81.4%	405	81.3%	1	1.02 (0.65–1.60)
No	48	18.3%	44	18.6%	92	18.5%		
Unreported	1	0.4%	0	-	1	0.2%		
Incarcerated								
Yes	220	84.0%	193	81.8%	413	82.9%	0.632	1.14 (0.71–1.82)
No	42	16.0%	42	17.8%	84	16.9%		
Unreported	0	-	1	0.4%	1	0.2%		
Unprotected Sex								
Yes	205	78.2%	183	77.5%	388	77.9%	1	1.02 (0.67–1.56)
No	57	21.8%	52	22.0%	109	21.9%		
Unknown	0	-	1	0.4%	1	0.2%		
Transactional Sex								
Yes	82	31.3%	66	28.0%	148	29.7%	0.38	1.21 (0.82–1.78)
No	174	66.4%	169	71.6%	343	68.9%		
Unknown	3	1.1%	1	0.4%	4	0.8%		
Unreported	3	1.1%	0	-	3	0.6%		
Unhoused								
Yes	191	72.9%	161	68.2%	352	70.7%	0.32	1.24 (0.84–1.83)
No	70	26.7%	73	30.9%	143	28.7%		
Unreported	1	0.3%	2	0.8%	3	0.6%		
Currently Use Drugs *								
Yes	200	76.3%	143	60.6%	343	68.9%	0.0002	2.09 (1.43–3.09)
No	62	23.7%	93	39.4%	155	31.1%		

* Indicates significance.

## Data Availability

The data presented in this study are available on request from the corresponding author due to the need to protect participant privacy.

## Supplementary Materials

The following supporting information is available at https://www.mdpi.com/article/10.3390/v16040628/s1: Table S1: Self-reported previous viral hepatitis A, B, C and D positive test results and association with active hepatitis B and delta infection; Table S2: Self-reported setting of receiving a tattoo and association with active hepatitis B infection; Table S3: Self-reported risk factor variables and odds ratios for the study sample, and association with HBcAb; Supplementary File S1: Participant Demographic Questions.
