# Supplementary material for: Dismantling Barriers to Hepatitis B and Delta Screening, Prevention, and Linkage to Care among the PWUD Community in Philadelphia
Source: Viruses. 2024 Apr 18;16(4):628. doi: 10.3390/v16040628 (PMC11054430; doi:10.3390/v16040628)
Supplement: Supplementary file 1 [file viruses-16-00628-s001.zip › viruses-2963123-supplementary.pdf]

Supplementary File S1: Participant Demographic Questions

PARTICIPANT INFORMATION	Participant Consent Date: __________<br>Participant Study ID: _________

PRIMARY LANGUAGE: □ English □ Spanish
DATE OF BIRTH: ____/_____/_____
PLACE OF BIRTH: □ USA □Other: ____________

RACE: □ Black □ White □ Asian – Country of Origin : __________ □ African □ Pacific Islander □ Native American □ Other _______________

HISPANIC □ Yes □ No □ Unk **GENDER:** □ Female □ Male □ Transgender □ FTM □ MTF

WHAT SERVICES ARE YOU SEEKING AT PPP: □ Exchange □ Drug Treatment □ HIV/HCV Testing □ Counselor □ Medical Care □None (here to hang out/rest) □ Other _____________

TESTING HISTORY

Have you ever <u>tested positive</u> for:

Hepatitis A Virus (HAV) □ Yes □ No □ Unk If yes, when? ___________
Hepatitis B Virus (HBV) □ Yes □ No □ Unk If yes, when? ___________
Hepatitis C Virus (HCV) □ Yes □ No □ Unk If yes, when? ___________
Hepatitis D Virus (HDV) □ Yes □ No □ Unk If yes, when? ___________

Have you ever been <u>vaccinated</u> for:

HAV □ Yes □ No □ Unk If yes, when? __________
HBV □ Yes □ No □ Unk If yes, when? __________

CLINICAL & RISK FACTORS

Have you ever:	Yes	No	Unk
Been incarcerated?	□	□	□
If yes, in the last 6 months?	□	□	□
Gotten a tattoo?	□	□	□
If yes, where: □ Tattoo parlor/shop □ Tattoo party □ Jail/Prison □ Friend/Relative □ Other __________			
Injected drugs? If yes, in the last 6 months? If yes, shared needles/works?	□	□	□
Had unprotected sex?	□	□	□
If yes, in the last 6 months?	□	□	□
Had sex for money or drugs?	□	□	□
	□	□	□

How old were you when you started injecting? ________
□ I don't inject drugs

What is your preferred method of drug consumption?
□ Injection □ Smoke □ Oral □ Sniff
□ Other _________ □ I don't consume drugs

Have your sexual partners in your lifetime been:
□ Male □ Female □ Both

Are you currently homeless, living on the streets, or living in a shelter? □ Yes □ No □ Unknown

Notes: ____
____
____
____
____

Table S1. *Self-reported previous viral hepatitis A, B, C and D positive test results and association with active hepatitis B and delta infection*

Category	HBsAg +		HBsAg -		Total		p-value	OR
	n	%	*n*	%	*n*	%		
HepA Previous Positive							1	-
Yes	0	-	17	3.5	17	3.4		
No	10	100	451	92.4	461	92.6		
Unkown	0		20	4.1	20	4.0		
HepA Vaccine							1	0.87
Yes	2	20	109	22.3	111	22.3		
No	5	50	235	48.2	240	48.2		
Unknown	3	30	144	29.5	147	29.5		
HepB Previous Positive							1	-
Yes	0	-	17	3.5	17	3.4		
No	10	100	447	91.6	457	91.8		
Unknown	0	-	24	4.9	24	4.8		
HepB Vaccine							.843	-
Yes	2	20	107	21.9	109	21.9		
No	4	40	238	48.8	242	48.6		
Unknown	4	40	143	29.3	147	29.5		
HepC Previous Positive							1	1.13
Yes	5	50	222	45.5	227	45.6		
No	5	50	251	51.4	256	51.4		
Unkown	0	-	15	3.1	15	3.0		
HepD Previous Positive							0.018	57.2
Positive	1	10	0	-	1	0.2		
Negative	8	80	488	100	496	99.6		
Unknown	1	10	0	-	1	0.2		

Table S2. *Self-reported setting of receiving a tattoo and association with active hepatitis B infection*

Category	HBsAg+		HBsAg -		Total		p-value	OR
	n	%	*n*	%	*n*	%		

Shop							0.69	0.68
Yes	5	0.6	281	70.8	286	70.6		
No	3	0.4	116	29.2	119	29.4		
Party				-			1	0
Yes	0	-	41	10.3	41	10.1		
No	8	100	356	89.7	364	89.9		
Jail							0.68	0.41
Yes	1	0.1	102	25.7	103	25.4		
No	7	0.9	295	74.3	302	74.6		
Friend/Family							0.71	1.6
Yes	4	50	155	39	159	39.3		
No	4	50	242	61	246	60.7		
Other							1	0
Yes	0	-	19	4.8	19	4.7		
No	8	100	378	95.2	386	95.3		

Note: OR & p-values reported based on those that have tattoos (n = 405).

Table S3. *Self-reported risk factor variables and odds ratios for the study sample, and association with HBcAb*

Category	HBcAb+ †		HBcAb-		Total		P-value	OR
	n	%	*n*	%	*n*	%		
Unhoused*								
Yes	65	61.3	287	73.2	352	70.7	0.04	-
No	40	37.7	103	26.3	143	28.7		
Unreported	1	0.9	2	0.5	3	0.6		
Transactional sex								
Yes	41	38.7	107	27.3	148	29.7	0.08	-
No	65	61.3	278	70.9	343	68.9		
Unreported	0	-	7	1.8	7	1.4		

† *Includes six individuals who also tested positive for HBsAg*

**Indicates significance*
